# The effects of lactic acid bacteria and yeasts as probiotics on the growth performance, relative organ weight, blood parameters, and immune responses of broiler: A meta-analysis

**DOI:** 10.12688/f1000research.51219.1

**Published:** 2021-03-05

**Authors:** Osfar Sjofjan, Danung Nur Adli, Rakhmad Perkasa Harahap, Anuraga Jayanegara, Dicky Tri Utama, Ainun Pizar Seruni

**Affiliations:** 1 Department of Nutrition and Feed Technology, University of Brawijaya, Malang, East Java, 65145, Indonesia; 2 Animal Feed and Nutrition Modelling (AFENUE) Research Group, IPB university, Bogor, West Java, 16680, Indonesia; 3 Study Program of Animal Science, Univeristy of TanjungPura, Pontianak, Borneo, 78124, Indonesia; 4 Department of Nutrition and Feed Technology, IPB University, Bogor, West Java, 16680, Indonesia; 5 Department of Animal Product Technology, Faculty of Animal Science, Malang, East Java, 65145, Indonesia; 6 Graduate School, University of Missouri, Missouri, Columbia, 65211, USA

**Keywords:** broiler, lactic acid bacteria, meta-analysis, probiotic, yeast.

## Abstract

**Introduction**: The number of publications in Scopus on this topic increased from less than 50 in 1995 to more than 250 in 2015. In other hand, Inconsistency in results about the correlation between yeast and lactic acid bacteria as probiotics has been evident since the early publications on use in broilers.

**Methods**: A meta-analysis was conducted to determine relationship between lactic acid bacteria and yeast as probiotics to broiler diets on the growth performance, relative organ weight, blood parameters, and immune response of the broiler.  A database was designed based on published data that reported the use of probiotics on the broiler. The method used for selecting articles was based on the Preferred Reporting Items for Systematic Review and Meta-Analyses (PRISMA) method. Articles selected were taken from PubMed, Web of science, Scopus, Google Scholar, and Science direct databases as well as individual.

**Results**: The final database consists of 49 *in vivo* articles, 93 studies, and 225 treatments. The analysis statement in the system was a PROC MIXED procedure of SAS software. The level of probiotic increased (p <0.001) body weight, body weight gain, and feed intake of broiler. There was a reduction (p <0.01) on feed conversion ratio and mortality on the level probiotic given to broiler. Supplementation of probiotics in broiler diet increased (p <0.001) the weight of liver, spleen, gizzard, bursa of fabricius and carcass yield, while reduced (p<0.001) abdominal fat weight. The probiotic given increased the total of red and white blood cells (both at p < 0.001) but did not affect lymphocyte.

**Discussion**: It can be concluded the yeast act as supporting agent that serves lactic acid bacteria as probiotic increases the growth performance, relative organ weight, blood parameters, and immune response of the broiler.

## Introduction

In 1997, the use of antibiotics in livestock was first addressed in Denmark with *avoparcin* as an antibiotic growth promoter (AGP). The trend continued and a European Union (EU)-wide ban on AGPs in animal feed (poultry) took effect in 2006 (EC Regulation No 1831/2003) ^1^. Since then, this type of regulation has spread to developing countries, including Indonesia, which has been banning antibiotics and imported poultry feed products since the most recent regulation, PERMENTAN/14/16/2017, was put in place ^2^. The EU introduced probiotics as an alternative to antibiotics and this has subsequently become an area of great interest for researchers worldwide ^3^. Probiotics are living microorganisms that when ingested in sufficient amounts, may positively improve growth, intestinal health and animal productivity. Probiotics are commonly sourced from lactic acid bacteria, namely, *Lactobacillus* and *Bifidobacterium*, which are usually found in the intestine ^4^.

Earlier studies have reported an active role for probiotics in reducing or eliminating the pathogen bacteria in the intestine. In recent research ^5, 6^ probiotic mixtures have also been found to have beneficial effects against a wide range of disorders, although evidence that mixtures are more effective than their component strains is more limited. Nevertheless, in the future, a further potential advantage of multi-strain probiotics, in addition to exerting additive or synergistic effects, is that the strain-specific effects of individual probiotic components could together exert a broader spectrum of activity ^5, 6^. Probiotics can be given in both powder and liquid form and positively modulate the composition of broiler intestinal microflora via the stimulation of potentially beneficial bacterial populations and the reduction of pathogenic bacteria ^1– 4^. The interaction between probiotics and micro biota added to diet influences the microbial population’s stability and the health of the host. The gut micro biota plays a crucial role in host metabolism and fundamentally influences physiology, health and well-being, functionality and performance ^5^.

Yeasts have been reported to act as supporting agents for lactic acid bacteria but also as having the potential to reduce avian bacterial in the gut micro biota of poultry ^1, 4^. Inconsistency in results about the correlation between yeast and lactic acid bacteria as probiotics has been evident since the early publications on use in broilers ^4^. Accordingly, the current study aims to determine the relationship between lactic acid bacteria and yeasts as probiotics in broiler diets on growth performance, meat quality, blood parameters, and immune responses, through a meta-analysis using data from published articles.

## Methods

### Database development

A database was constructed based on peer-reviewed and published research articles which reported the use of probiotics in the broiler diet. Articles were selected based on the Systematic Review Center for Laboratory Animal Experimentation (SYRCLE') method ^7^ and Preferred Reporting Items for Systematic Review and Meta-Analyses (PRISMA) ^8^. Articles selected were taken from PubMed, Web of science, Scopus, Google Scholar, and Science direct databases as well as individual journals such as World Poultry Journal Science, British Poultry Science Journal, and International Journal of Poultry Science using the keywords ‘probiotic’, ‘broiler’, ‘performance’, ‘organ weight’, ‘carcass’, and ‘blood serum’. In each article evaluated, the reference list was also searched for relevant articles. The raw database information from articles, authors, year of study, broiler (strain and sex), diet used in trial, length of trial, level of treatment, form and dosage of probiotic contained in the study was recorded in a spreadsheet following the referenced method ^7^. The parameters included were growth performance, relative organ weight, carcass quality, blood parameters, and immune responses. The strains recorded on the raw database were Ross308, which dominated at 63.26%; Arbor Acres at 32.65%; and others at 4.09%. 

Criteria for an article to be included in database were as follows: (a) article was published in a peer-reviewed with range 2008–2020, this paper length was chosen as related to journals aged last 10–12 years are often good ^9^ (b) the broiler were modern-controlled-trial environment and management, (c) performed directly on broiler *in vivo* as the experimental animals, (d) The log concentration of lactic acid bacteria and yeast both powder and liquid form on the trial was transformation into 10 ^10^ in the database development, (e) non-probiotic treatment excluded from the database, (e) the articles written consistently in English were considered in studies, (f) the average duration of the study was minimum 0–21 days and the maximum at 0–53 days, (g) dosages given at the range 0–10 g/kg from total feed formulation. Moreover, the dependent and independent variables were selected with the aim of lactic acid bacteria and yeast related as probiotic on the broiler. Likewise, data extraction was completed in accordance with the task analysis to obtain the exact values from graphical data, the relevant figure from the papers were subjected to an online tool, WebplotDigitizer 4.4 ( https://automeris.io/WebPlotDigitizer/), following the method ^10^.

**Figure 1.**
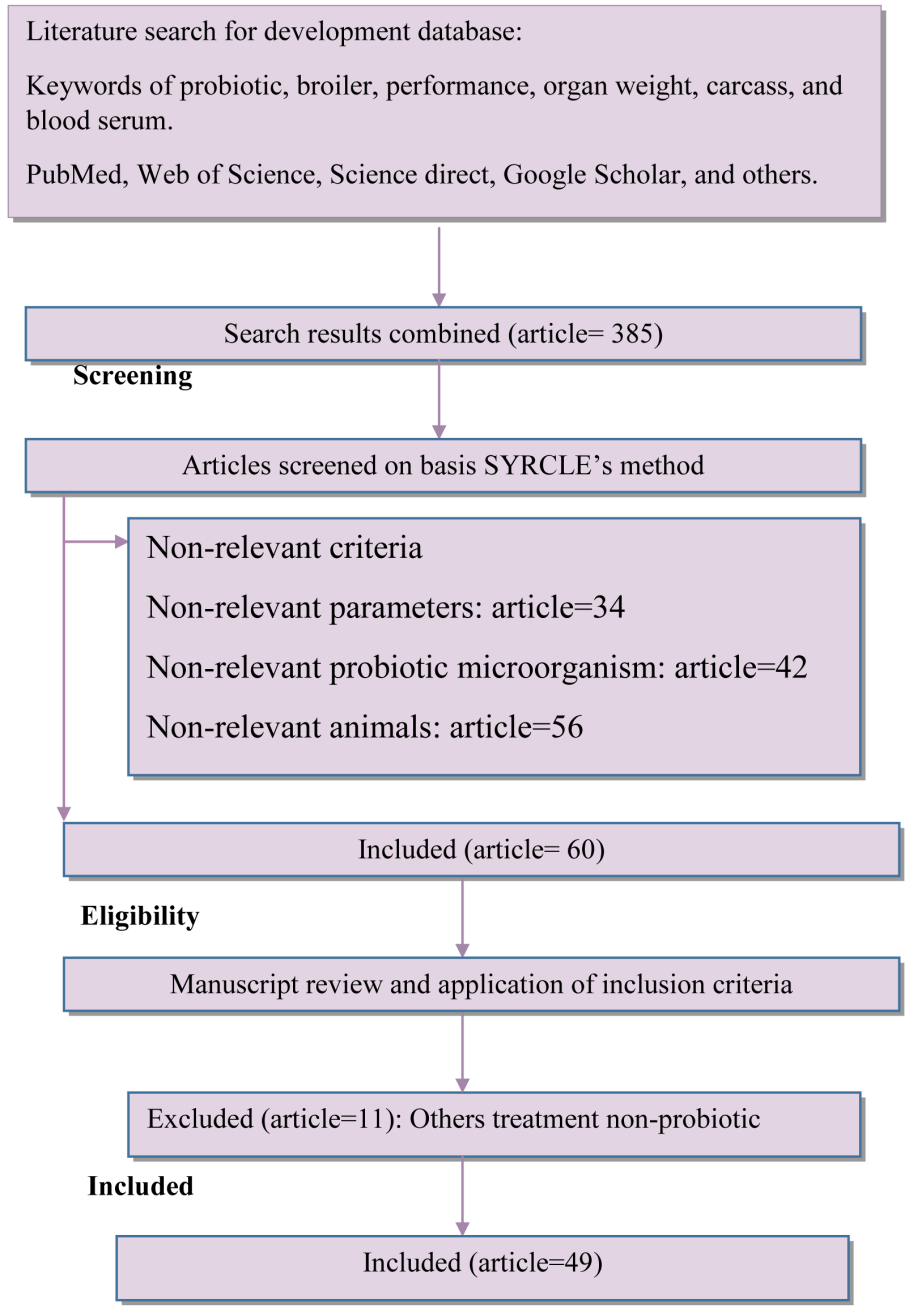
Diagram flow of article selection included in meta-analysis adapted from PRISMA Method.

The final database consisted of 49 *in vivo* articles, 93 studies, and 225 n-total (3,375 n-total of total in this experiment). The details for the study selection included in this meta-analysis are provided in Figure 1. The search strategy is presented in Table 1. The summary of the final database is presented in Table 2, and PICOS criteria presented in Table 6.

**Table 1.** Search strategy.

Databases	Search strategy
PubMed	((("probiotics" [MeSH Terms] OR "probiotics" [All Fields] OR "probiotic" [All Fields]) OR (serum [All Fields] AND blood [All Fields ] AND blood [All Fields])) OR
Scopus	("broiler" [MeSH Terms] OR "broiler" [All Fields])) OR (organ [All Fields] AND ("weight" [Journal] OR "weight" [All Fields ]))) AND ("chickens" [MeSH Terms] OR "carcass" [All Fields] OR "carcass" [All Fields]).
Google Scholar	‘Probiotic’ ‘Growth Performances’ ‘Relative organ weight’ ‘Blood performances’ ‘Carcasses’ ‘broiler’ ‘Cited by’ ‘related articles’ ‘Since year’ ‘2008–2020’ Search in full text Search in title Result 1 Result 1 Result 2 Result 2 Result 3 Result 3 Result 4 Result 5 Result 5 Result 6 Result 6 Result 7 Result 7 Result 8

**Table 2.** Studies included in the meta-analyses of the relationship between lactic acid bacteria and yeast as probiotics on the growth performance, relative organ weight, blood parameters, and immune response of broiler.

No	References	Kind of Probiotic	Form	Dosage (g/kg)	Periods (d)
1	Chen *et al.* ^18^	Lactic acid bacteria	Powder	0-2	0-28
2	Park and Kim ^19^	Lactic acid bacteria	Powder	0-1	0-28
3	Zhang *et al.* ^20^	Lactic acid bacteria	Powder	0-1	0-35
4	Jamshidparvar *et al.* ^21^	Lactic acid bacteria	Liquid	0-2	0-42
5	Khan *et al.* ^22^	Lactic acid bacteria	Liquid	0-1	0-39
6	Gheisar *et al.* ^23^	Lactic acid bacteria	Powder	0-0.50	0-35
7	Hussein *et al.* ^24^	Yeast	Powder	0-0.50	0-35
8	Nosrati *et al.* ^25^	Lactic acid bacteria	Powder	0-0.18	0-42
9	Javandel *et al.* ^26^	Lactic acid bacteria	Powder	0-0.9	0-42
10	Sun and Kim ^27^	Yeast	Powder	0-0.02	0-35
11	Sugiharto *et al.* ^28^	Lactic acid bacteria	Powder	0-0.05	0-42
12	Ghasemi *et al.* ^29^	Lactic acid bacteria	Powder	0-0.04	0-42
13	Abdel-Hafeez *et al.* ^30^	Lactic acid bacteria	Powder	0-1.5	0-42
14	Toghyani *et al.* ^31^	Lactic acid bacteria	Powder	0-0.15	0-42
15	Salah *et al.* ^32^	Lactic acid bacteria	Powder	0-2	0-42
16	Paryad and Mahmoud ^33^	Yeast	Powder	0-0.02	0-42
17	Koc *et al.* ^34^	Yeast	Powder	0-2	0-21
18	Zhou *et al.* ^35^	Lactic acid bacteria	Powder	0-0.4	0-35
19	Rezaeipour ^36^	Yeast	Powder	0-7.5	0-42
20	Cho *et al.* ^37^	Lactic acid bacteria	Powder	0-0.2	0-35
21	Priya and Babu ^38^	Yeast	Powder	0-1.5	0-36
22	Lan *et al.* ^39^	Lactic acid bacteria	Powder	0-0.05	0-35
23	Sun and Kim ^40^	Yeast	Powder	0-0.2	0-35
24	Elnagar ^41^	Yeast	Powder	0-0.2	0-53
25	Mashayekhi *et al.* ^42^	Lactic acid bacteria	Powder	0-0.5	0-42
26	Attia *et al.* ^43^	Yeast	Powder	0-1	0-35
27	Pournazari *et al.* ^44^	Lactic acid bacteria	Powder	0-2	0-42
28	Riyazi *et al.* ^45^	Lactic acid bacteria	Powder	0-0.15	0-42
29	Sugiharto *et al.* ^46^	Yeast	Powder	0-0.4	0-35
30	Manafi *et al.* ^47^	Yeast	Powder	0-0.01	0-42
31	Ashayerizadeh *et al.* ^48^	Lactic acid bacteria	Powder	0-0.9	0-42
32	Zahirian *et al.* ^49^	Yeast	Powder	0-4	0-42
33	Sugiharto *et al.* ^50^	Lactic acid bacteria	Powder	0-0.2	0-38
34	Sugiharto *et al.* ^51^	Lactic acid bacteria	Powder	0-0.15	0-35
35	Sugiharto *et al.* ^52^	Lactic acid bacteria	Powder	0-0.01	0-35
36	Miah *et al.* ^53^	Lactic acid bacteria	Liquid	0-0.5	0-21
37	Sherief *et al.* ^54^	Yeast	Powder	0-0.5	0-42
38	Reisinger *et al.* ^55^	Yeast	Powder	0-0.2	0-35
39	Attia *et al.* ^56^	Yeast	Powder	0-0.05	0-35
40	Sjofjan and Adli ^57^	Lactic acid bacteria	Combination	0-0.8	0-35
41	Makled *et al.* ^58^	Lactic acid bacteria	Powder	0-5	0-42
42	Vase *et al.* ^59^	Lactic acid bacteria	Liquid	0-0.03	0-42
43	Waqas *et al.* ^60^	Yeast	Powder	0-0.06	0-35
44	Caruk *et al.* ^61^	Lactic acid bacteria	Powder	0-0.1	0-42
45	Yalçin *et al.* ^62^	Yeast	Powder	0-3	0-42
46	Yalçinkaya *et al.* ^63^	Yeast	Powder	0-1	0-42
47	Shokaiyan *et al.* ^64^	Lactic acid bacteria	Powder	0-0.5	0-42
48	Salehizadeh *et al.* ^65^	Lactic acid bacteria	Powder	0-0.1	0-42
49	Khajeh *et al.* ^66^	Lactic acid bacteria	Powder	0-0.5	0-42

### Data analysis

Statistical dataset analysis using a mixed-model approach was applied ^11– 14^ with statement analysis in the system using the MIXED procedure of SAS (version 9.1, SAS Institute Inc., 2008), the following model was applied: The findings of a study was then taken as a random effect, while the supplementation concentration was taken as the fixed effect as follows ^15– 17^:

$$Y_{ij}=B_{0}+B_{1}X_{ij}+B_{2}{X^{2}}_{\text{ij}}+s_{i}+b_{i}X_{ij}+e_{ij}$$

where Yij = the expected output for dependent variable Y at level j from the variable X as a continuous

variable in the study *i*,, B _0_ = overall intercept across all studies (fixed effect), B _1_ = linear regression coefficient of Y on X (fixed effect), B _2_ = quadratic regression coefficient of Y on X (fixed effect), X _ij_ = value of the continuous predictor variable (probiotic supplementation level), s _i_ = random effect of study i, b _i_ = random effect of study i on the regression coefficient of Y on X in study i and e _ij_ = unexplained residual error. In the statement CLASS, the “study” variable was declared. Data were weighted by the number of replicates in each study. Additionally, an unstructured variance – covariance matrix (type = un) was performed at the random effect part of the model to avoid a positive correlation between intercepts and slopes. Significance of an effect was stated at the probability level of *p* < .05, and *p* < .1 was considered as a tendency of significance. In case that the quadratic model above was not significant, the model was changed into its corresponding linear model. The variable of the study was declared in the class statement as it did not contain any quantitative information. The regression equations are also presented with *p-*value, and root mean square error (RMSE).

Furthermore, to determine (1) interaction between lactic acid bacteria and yeast; (2) interaction of type probiotic (powder and liquid) according to the following model ^16^:

Yij=μ+Si+τj+Sτij+eij

Where Y *ij* = the expected output for dependent variable *Y,* μ = overall mean, *Si* = random effect of *I* study, *τj* = fixed effect of the *j* level, *Sτij* = random interaction between *i* study and the *j* level, and *eij* =residual error. A significant effect was declared at p<0.05 or there is a tendency when the p-value was between 0.05 and 0.10.

## Results

Table 3 presents the effects of probiotics on broiler performance. The meta-analysis results show the level of probiotic (p<0.001) body weight, body weight gain, and feed intake of broilers. In contrast, there was a reduction (p <0.01) on feed conversion ratio (FCR) and mortality on the level probiotic given to broiler. Furthermore, the analysis also shows that the form of probiotic in the feed does not create any significant difference in broiler performance.

**Table 3.** Effect of probiotics on performance of broiler.

			Parameter estimates				Model statistics			
Response parameter	Unit	n	Intercept	SE intercept	Slope	SE slope	*p*-Value	RMSE	P x L	B x Y
BW	gram	225	2071	62.1	13.1	2.22	<.001	314	0.523	0.166
BWG	gram	225	1567	61.7	13.8	1.76	<.001	248	0.506	0.847
FCR	-	225	1.85	0.031	-0.010	0.00	<.001	0.193	0.630	0.310
FI	gram	225	3163	120	4.74	2.7	<.001	380	0.609	0.361
Mortality	%	225	0.665	0.179	-0.002	0.01	<.001	1.80	0.83	0.474

Note: P = powder; L=liquid; B=LAB; Y=yeast; Slope: The respond when the probiotic at the zero level, SE intercept: standard error intercept; BW: body weight; BWG; body weight gain; FCR: feed conversion ratio; FI; feed intake; root mean square error (RMSE)

The weight of abdominal organs and carcass yield of broilers were affected by the supplementation of probiotics in the diet ( Table 4). Supplementation of probiotics in broiler diet increased (p <0.001) the weight of liver, spleen, gizzard, bursa of Fabricius and carcass yield, while reduced (p<0.001) abdominal fat weight. Different types of probiotic, i.e., powder or liquid, influenced the weight of liver (p =0.001), spleen (p <0.005), gizzard (p =0.045) and bursa of Fabricius, (p <0.001). In contrast, abdominal fat and carcass yield were not affected by the type of probiotics supplemented in the diet. Further, different culture type, i.e., lactic acid bacteria or yeast, had no significant effect on the abdominal organs weight and carcass yield of broilers. 

**Table 4.** Effect of probiotics on carcass and organ weight of broiler.

			Parameter estimates				Model statistics			
Response parameter	Unit	n	Intercept	SE intercept	Slope	SE slope	*p*-Value	RMSE	P x L	B x Y
Liver	g/kg	225	2.44	0.06	0.003	0.004	<.001	0.60	0.0001	0.27
Spleen	g/kg	225	0.34	0.07	0.004	0.002	<.001	0.30	0.0005	0.11
Gizzard	g/kg	225	1.50	0.06	0.003	0.002	<.001	0.26	0.004	0.52
Bursa of fabricius	g/kg	225	0.34	0.055	0.0005	0.002	<.001	0.34	<.001	0.51
Abdominal fat	g/kg	225	1.58	0.07	-0.015	0.004	<.001	0.62	0.37	0.50
Carcass yield	%	225	67.6	0.73	0.067	0.02	<.001	3.36	0.38	0.23

Note: P= powder; L=liquid; B=LAB; Y=yeast; Slope: The respond when the probiotic at the zero level, SE intercept: standard error intercept; root mean square error (RMSE)

The effects on blood parameters of lactic acid bacteria and yeasts as probiotics are presented in Table 5. The probiotic given increased the total of red and white blood cells (both at p < 0.001) but did not affect lymphocyte. Furthermore, the immune response hemoglobin results were not significantly influenced by the delivery of different lactic acid bacteria and yeast forms such as powder or liquid.

**Table 5.** Effect of probiotics on blood and immune responses of broiler.

			Parameter estimates				Model statistics			
Response parameter	Unit	n	Intercept	SE intercept	Slope	SE slope	*p*-Value	RMSE	P x L	B x Y
RBC	/ μL	225	2.25	0.05	0.003	0.002	<.001	0.30	0.38	0.50
WBC	/ μL	225	289	9.40	0.42	0.41	<.001	58	0.21	0.03
Limphocyte	%	225	53	1.55	-0.02	0.06	<.001	8.73	0.47	0.004
Hemoglobin	mg/dL	225	8.24	0.38	0.003	0.01	<.001	1.63	0.70	0.97

Note: RBC: red blood cell; WBC: white blood cell; P= powder; L=liquid; B=LAB; Y=yeast; Slope: The respond when the probiotic at the zero level, SE intercept: standard error intercept; root mean square error (RMSE)

**Table 6.** PICOS Criteria.

	Search strategy	Exclusion criteria
Participant	Broiler	Non-broiler
Interventions	Probiotic (Lactic acid bacteria and yeast)	Irrelevant treatment
Comparison	Control group (Maize – Soya bean diet [basal])	
Outcomes	LAB, BW, BWG, FCR, FI, RBC, WBC, RMSE, SE, PXL	
Study design	Modern controlled environment during *in-vivo*	

P = powder; L=liquid; B=LAB; Y=yeast; Slope: The respond when the probiotic at the zero level, SE intercept: standard error intercept; BW: body weight; BWG; body weight gain; FCR: feed conversion ratio; FI; feed intake; RBC: red blood cell; WBC: white blood cell root mean square error (RMSE); PICOS: population, intervention, comparison, outcomes and study

## Discussion

### The effect of probiotics on growth performance

Our meta-analysis shows that probiotics positively affect growth performance. In terms of growth performance, we suggest that this finding is related to the ability of probiotics to induce intestinal mechanisms, resulting in a reduction in pathogenic bacteria. In the digestive system, intestinal pH, intestinal bacteria composition, and digestive activity are improved when probiotics are present in diets ^18^. Some probiotics are known to produce enzymes, amylase, protease, and lipase to optimize nutrients’ breakdown ^19, 20^. They can also increase specific enzymes in the host digestive tract to enhance nutrient absorption in the diet. In the poultry industry, probiotics are supplemented into the diet to maintain health by enhancing gut health, modulating the immune system, lowering glycaemic response, and improving various performances parameters ^21– 23^. Moreover, the administering of probiotics has several ways in practice. The administering of probiotics can be included in the basal diet or combined with raw materials that contain prebiotics to enhance its effect ^20, 30, 50– 54, 67^. The probiotic can be given alone or with another additive without any negative effect such as acidifiers and phytogenics ^25, 26, 32, 42, 44^. Furthermore, probiotics can contain one or multiple microorganism strains that can be added to animals' diets ^24, 28, 37^. 

There are previous studies that report that cell-wall components of yeast in dietary supplementation to lactic acid bacteria improve the growth rate, feed consumption, and feed efficiency in broilers ^51, 55^. These positive and consistent results were due to yeast activating spores to reduce and remove potential pathogens in the gut which possibly increases body weight ^44^. Linearly, the factors related to synergism between yeast and lactic acid bacteria reported in the research could be related to environmental conditions in various experiments ^45, 46^.

One study from 46 showed that rearing the broiler with a heat-stress environment at 35 ^0^c was more low weight than heat stress exposed. Thus, yeast failed to alleviate heat-stress on the performance of broiler ^46^. The lower temperature in the chicken house may help increase feed intake to eat more of the experimental diets ^57^. In addition, other factors related due to being reared under a stocking density stress of 43 kg live weight per m ^2^ floor space ^57^. Another result ^45^ explains in more detail that yeasts in powder form significantly increase the body weight of broilers starting at 21 days old, with an increase in line with increasing feed intake and reducing FCR. These consistent results ^57– 59^ explain that the use of lactic acid bacteria as probiotics in powder form help to increase the body weight of broilers, as a result of digestibility and metabolic process improvement caused by the bacteria, affecting energy partition and putting more energy into growth than maintenance ^46 ^.

Moreover, the positive use of probiotics both as powders and liquids was in line with the increasing level of treatment in broiler ^57^. The probiotics enhance liquid lactic acid bacteria synergism with yeast in the feed but suggested at an optimized level ^57^ of 0.8%. However, the dose-response relationship of probiotics in animal trials is rarely studied ^1^. At low doses a probiotic may be specific, for example *bifidobacteria*, due to the high specificity of *bifidobacteria* for that particular probiotic ^1^. In other hand, if the dose increased, this would leave some substrate for other probiotic strains able to ferment it. The outcome of high dose would show less specificity that that of the low dose ^1^. Treatment with both powder and liquid forms increases body weight and feed intake and reduces feed conversion ^31, 57, 61^. Moreover, probiotics for farm animals have positive effects on growth, efficiency of feed utilization ^68^. In addition, the consistent result in studies ^45^vs 50– 52 show the relationship between both yeast and lactic acid bacteria working together to reduce potential pathogens in the gut of broilers but dose is dependent.

### The effect of probiotic on the relative organ weight and carcass quality

The meta-analysis results show limited effects on the carcass and organ weight of broilers. In agreement from 52, 62 the carcass quality shows no significant difference after administering probiotics of both lactic acid bacteria and yeast. The one factor can be caused reduced of percentage carcass are heat-stress environmental. The carcass heat-stress was associated with the reduced of carcass quality ^52^. Apart from physiological adjustment derived from depressed feed intake, the increased *Corticosterone* level may be responsible lower percentage of carcass ^52^. Carcass percentage was reported to increase by one study ^45^, with the saleable product in terms of edible portions. Reported ^45^ the carcass quality can be affected from physiological and genetic potential, feed formulated, strain of the broiler rearing. The excess fat deposition in carcass of broiler is undesirable to producers because of reduced carcass yield and to consumers that prefer a leaner product ^1^.

Likewise, one study ^62^ the use of yeast as probiotic reduced abdominal fat of broiler. Reported ^62^ yeast help to reduce fat deposition because, modern-broiler-farming were intensive feeding ( *ad-libitum*), fatter caused limb defects, and sudden death syndrome. The probiotics reported reduced fat of broiler compared without probiotic ^65^. The mode of action was that probiotics decreased the activity of acetyl-CoA carboxylase ^66^. Acetyl-CoA carboxylase has been widely suggested as the rate-limiting enzyme in fatty acid synthesis ^66^. The decline in the synthesis of fatty acids, in turn, would decrease their availability for esterification to triglycerides for deposed in the adipose tissue ^66^. Furthermore, the minimum dose of probiotic to stimulate fatty acids are currently unknown ^63, 64, 66^. Differences in the broiler line/breed and conditions, as well as microorganism strains (highly species-and strain specific), origin species, concentrations, and methods of administration of the probiotic bacteria, may explain these results ^65^. However, our study can’t exactly suggest the dose optimum for using this probiotic.

In one study ^42^, the effects of probiotics on relative weights of liver and spleen were not significant (P > 0.05), while bursa of Fabricius relative weight increased (p < 0.05). Supplementing diets with probiotics could help to prevent necrotic enteritis which associated with degeneration of hepatocytes and immune system of the broiler ^69^. The smaller liver in broiler may indicate a higher resistance to pathogen microorganism such as *Clostridium perfrigens* ^70^. The IGF-1 can produce short-fatty acids (SCFAs), which act either directly or indirectly on the liver and adipose tissue to promote growth of organ and skeletal development ^65^. The report from ^71^at the end of the feeding trial showed that the development of gizzard weight was decreased, dateable irregularities in the gizzard are a sensitive index to reduce anti-nutritional factors in the basal diet after exposure to toxic substance not to amount of lactic bacteria ^71^. Moreover, increased weight of this lymphoid organ may indicate a higher immunity achieved in treated broiler, which could be explained by probiotic anti-microbial activity ^18, 42^. The factor affected by significant differences in the relative organ weight is the ability to absorb substances from probiotics ^18, 43, 44^. 18 stated that variances between the broilers result from impacts on absorption and other capacities of the relative organ weight. The growth factors correlate with age, while the broiler's uses in the relative age cause the same internal organ’s growth. In instances, an increased relative organ weight may be in line with an increase in lymphocyte concentration ^18^.

### The effect of probiotic on the blood parameters and immune responses

The meta-analysis of different probiotic levels on some blood parameters showed red and white blood cell concentration increased ( *p* <0.05) with increasing probiotic supplementation levels in the feed. The increased of the white blood cells had correlated with yeast reduce the uric acid (UA) content in the blood ^72^. The uric acid is a metabolite of protein that has an antioxidant function, but is converted to a pro-oxidant in the cell or cytoplasm ^72^. In contrast, lymphocyte, and hemoglobin were not significantly different ( *p* >0.05) ^15^. Linearly, blood serum rose in line with probiotic increase. Additionally, one ^18^ shows that, the lactic acid bacteria that help reduce avian-pathogenic bacteria were *Escherichia coli* and *Clostridium perfrigens*. The beneficial action indicates that lactic acid bacteria produced extracellular enzymes to enhance the nutrient digestibility of feed and synthesize immune function using endogenous anti-microbial ^20^. In terms of negative linear response in studies ^21^ there was no positive result on red and white blood cells.

Moreover, probiotics could be related to a lowered recycling of bile salts in the gut or inhibited hepatic 3-hydroxy-3-methylglutaryl coenzyme A reductase activity ^21^. The mechanism operating in lactic acid bacteria, as probiotics to elicit their hypocholesterolemic effect is interference with intestinal bile acid transport and absorption, leading to an increase in bile acid excretion ^21^. The potential pathogens reduce but are not eliminated, thus, probiotics balance the intestinal environment to enhance the broiler’s immune systems ^45, 50– 52^. Although, the lactic acid bacteria do not produce butyric acid themselves, they stimulate the proliferation of butyric acid and cell-wall of yeast in the blood circulation by the mechanism that is called cross-feeding ^65^. Continued research ^42 ^shows probiotics help to increase the white blood cell count as level of probiotic is increased. One study ^42^ stated that the increase of white blood cells and immune response was due to the level increase of B and T lymphocyte production. In line with the 41 studies the amount of red blood cells, hemoglobin, and white blood cells consistently tends to increase compared to controls. The positive effect from yeast as a probiotic could derive from its outer cell wall components namely: chitin, mannan, and glucan which have an immunostimulant effect. Moreover, these outer wall components promote lactic acid bacteria activity, which is activated by producing enzymes that cause disintegration of bile salts, making them unconjugated ^68^. The yeast can enhance the immune response by promoting growth of lactic acid bacteria and thus simultaneously producing antibacterial substances and stimulating the production of immunoglobulin ^33^. Thus, yeast acts as a supporting agent of lactic acid bacteria, which adhere to the endogenous epithelial cells to initiate colonization ^33^.

## Conclusions

The results provided by this meta-analysis demonstrates the enhancement of overall performance of broilers supplemented with lactic acid bacteria and yeast as probiotics. Effects of the probiotics on blood parameters are dose dependent, where areas, the additives have limited effects on organ weight and carcass percentage. Both powder and liquid forms of probiotics do not affect the results differently. The future research trends are to determine the dose optimum of probiotic for broiler.

## Data availability

### Underlying data

All data underlying the results are available as part of the article and no additional source data are required.

### Extended data

Figshare: Extended data for ‘The effects of lactic acid bacteria and yeasts as probiotics on the growth performance, relative organ weight, blood parameters, and immune responses of broiler: A meta-analysis’. https://doi.org/10.6084/m9.figshare.14060414 ^73^.

This project contains extracted data of outcome measures (BW: body weight; BWG; body weight gain; FCR: feed conversion ratio; FI; feed intake; RBC: red blood cell; WBC: white blood cell).

### Reporting guidelines

Figshare: PRISMA checklist for ‘‘The effects of lactic acid bacteria and yeasts as probiotics on the growth performance, relative organ weight, blood parameters, and immune responses of broiler: A meta-analysis’’. https://doi.org/10.6084/m9.figshare.14060501 ^74^.

Data are available under the terms of the Creative Commons Attribution 4.0 International license (CC BY 4.0).
