# Supplementary material for: The effects of lactic acid bacteria and yeasts as probiotics on the growth performance, relative organ weight, blood parameters, and immune responses of broiler: A meta-analysis
Source: F1000Res. 2021 Oct 13;10:183. Originally published 2021 Mar 5. [Version 3] doi: 10.12688/f1000research.51219.3 (PMC8567689; doi:10.12688/f1000research.51219.3)
Supplement: Peer review history

# Peer review: Reviewer response for version 3

**DOI:** 10.5256/f1000research.77954.r96902

**Published:** 2021-11-03

**Authors:** Youssef A. Attia

**Affiliations:** 1 Department of Agriculture, Faculty of Environmental Sciences, King Abdulaziz University, Jeddah, Saudi Arabia

I approve the changes made by authors and the manuscript may be indexed in the current form. There is an improvement in the contents and better illustration.

Are the rationale for, and objectives of, the Systematic Review clearly stated?

Yes

Is the statistical analysis and its interpretation appropriate?

Yes

Are sufficient details of the methods and analysis provided to allow replication by others?

Yes

Are the conclusions drawn adequately supported by the results presented in the review?

Partly

Reviewer Expertise:

Poultry production and nutrition

I confirm that I have read this submission and believe that I have an appropriate level of expertise to confirm that it is of an acceptable scientific standard.

# Peer review: Reviewer response for version 2

**DOI:** 10.5256/f1000research.76610.r93793

**Published:** 2021-09-23

**Authors:** Youssef A. Attia

**Affiliations:** 1 Department of Agriculture, Faculty of Environmental Sciences, King Abdulaziz University, Jeddah, Saudi Arabia

This MS has an updated review on the use of lactic acid bacteria and yeasts as probiotics on the growth performance, relative organ weight, blood parameters, and immune responses of broiler: A meta-analysis of value to broilers industry. The MS has provided helpful information about the concept of probiotics and their role in enhancing performance, health and product quality.

**Major concern**

- To go one step further, the dose of lactic acid bacteria and yeasts should be estimated based on this work.
- The update and renewable application of lactic acid bacteria and yeasts in broilers should given considered attention such as: compact of heat stress; control of infectious diseases; and detoxification of nitrate and aflatoxin. I have included some of my works which are good examples:
  - Control of *Salmonella enteritidis* infection in male layer chickens by acetic acid and/or prebiotics, probiotics, and antibiotics ^1^.
  - The detoxication of nitrate by two antioxidants or a probiotic, and the effects on blood and seminal plasma profiles and reproductive function of New Zealand White rabbit bucks ^2^.
  - Enhancing Tolerance of Broiler Chickens to Heat Stress by Supplementation with Vitamin E, Vitamin C and/or Probiotics ^3^.
  - Nitrate detoxification using antioxidants and probiotics in the water for rabbits ^4^.
  - Health Benefits of Probiotics in Human and Animal Nutrition and Prospective Research.
  - Effects of different dietary levels of blue lupine ( *Lupinus angustifolius*) seed meal with or without probiotics on the performance, carcass criteria, immune organs, and gut morphology of broiler chickens ^5^.

**Minor concern**

- Improve the language as the MS needs an English language and grammar edit.
- Enhancing the MS with figures and infographics can improve readability of the MS.

Are the rationale for, and objectives of, the Systematic Review clearly stated?

Yes

Is the statistical analysis and its interpretation appropriate?

Yes

Are sufficient details of the methods and analysis provided to allow replication by others?

Yes

Are the conclusions drawn adequately supported by the results presented in the review?

Partly

Reviewer Expertise:

Poultry production and nutrition

I confirm that I have read this submission and believe that I have an appropriate level of expertise to confirm that it is of an acceptable scientific standard, however I have significant reservations, as outlined above.

## References

[1] : Control of Salmonella enteritidis infection in male layer chickens byacetic acid and/or prebiotics, probiotics and antibiotics. *Arch.Geflügelk* .2012;76(4) :

[2] : The detoxication of nitrate by two antioxidants or a probiotic, and the effects on blood and seminal plasma profiles and reproductive function of New Zealand White rabbit bucks. *Animal* .2013;7(4) : 10.1017/S1751731112002054 591-601 10.1017/S1751731112002054 23177268

[3] : Enhancing Tolerance of Broiler Chickens to Heat Stress by Supplementation with Vitamin E, Vitamin C and/or Probiotics. *Annals of Animal Science* .2017;17(4) : 10.1515/aoas-2017-0012 1155-1169 10.1515/aoas-2017-0012

[4] : Nitrate detoxification using antioxidants and probiotics in the water for rabbits. *Revista Colombiana de Ciencias Pecuarias* .2018;31(2) : 10.17533/udea.rccp.v31n2a06 130-138 10.17533/udea.rccp.v31n2a06

[5] : Effects of Different Dietary Levels of Blue Lupine (Lupinus angustifolius) Seed Meal With or Without Probiotics on the Performance, Carcass Criteria, Immune Organs, and Gut Morphology of Broiler Chickens. *Front Vet Sci* .2020;7: 10.3389/fvets.2020.00124 124 10.3389/fvets.2020.00124 32232061PMC7082746

# Peer review: Reviewer response for version 2

**DOI:** 10.5256/f1000research.76610.r92326

**Published:** 2021-08-26

**Authors:** Hazem Mohammed Ebraheem Shaheen

**Affiliations:** 1 Department of Pharmacology, Faculty of Veterinary Medicine, Damanhour University, Damanhour, 22511, Egypt

No additional comments.

Are the rationale for, and objectives of, the Systematic Review clearly stated?

Partly

Is the statistical analysis and its interpretation appropriate?

Partly

Are sufficient details of the methods and analysis provided to allow replication by others?

Partly

Are the conclusions drawn adequately supported by the results presented in the review?

Yes

Reviewer Expertise:

Veterinary Pharmacology.

I confirm that I have read this submission and believe that I have an appropriate level of expertise to confirm that it is of an acceptable scientific standard.

# Peer review: Reviewer response for version 1

**DOI:** 10.5256/f1000research.54362.r90146

**Published:** 2021-07-26

**Authors:** Hazem Mohammed Ebraheem Shaheen

**Affiliations:** 1 Department of Pharmacology, Faculty of Veterinary Medicine, Damanhour University, Damanhour, 22511, Egypt

Please specify what is the object of this study.

 You could add an Economic interpretation of this study.

 Data in the Results section were insufficient and need more clear for discussion.

 This paragraph is not understandable:

*"Criteria for an article to be included in database were as follows: (a) article was published in a peer-reviewed with range 2008–2020, this paper length was chosen as related to journals aged last 10–12 years are often good9 (b) the broiler were modern-controlled-trial environment and management, (c) performed directly on broiler in vivo as the experimental animals, (d) The log concentration of lactic acid bacteria and yeast both powder and liquid form on the trial was transformation into 1010 in the database development, (e) non-probiotic treatment excluded from the database, (e) the articles written consistently in English were considered in studies, (f) the average duration of the study was minimum 0–21 days and the maximum rom total feed formulation. Moreover, the dependent and independent variables were selected with the aim of lactic acid bacteria and yeast related as probiotic on the broiler. Likewise, data extraction was completed in accordance with the task analysis to obtain the exact values from graphical data, the relevant figure from the papers were subjected to an online tool, WebplotDigitizer 4.4 (https://automeris.io/WebPlotDigitizer/), following the method10".*

 I suggest an English language edit for the revision of the manuscript.

Are the rationale for, and objectives of, the Systematic Review clearly stated?

Partly

Is the statistical analysis and its interpretation appropriate?

Partly

Are sufficient details of the methods and analysis provided to allow replication by others?

Partly

Are the conclusions drawn adequately supported by the results presented in the review?

Yes

Reviewer Expertise:

Veterinary Pharmacology.

I confirm that I have read this submission and believe that I have an appropriate level of expertise to confirm that it is of an acceptable scientific standard, however I have significant reservations, as outlined above.

# Response

**Published:** 2021-07-26

**Authors:** Danung Nur Adli

**Affiliations:** University of Brawijaya, Indonesia

We would like to express our sincere thanks and appreciation for comprehensively reviewed our manuscript that helped us to improve our manuscript.

 Regarding the reviewer suggestion and question

 1. Please specify what is the object of this study.

**Answer: **We already mentioned in the introduction that "Accordingly, the current study aims to determine the relationship between lactic acid bacteria and yeasts as probiotics in broiler diets on growth performance, meat quality, blood parameters, and immune responses, through a meta-analysis using data from published articles".

 2.You could add an Economic interpretation of this study.

**Answer: **Thank you for the suggestion. Regarding this suggestion the economic parameters, initially we input this parameter but in final paper we don't put down this parameter due to lack of data provided in the paper.

 3. Data in the Results section were insufficient and need more clear for discussion.

**Answer:** We mentioned clearly in the result section.

 4. **Answer: **The statement that you are asking for are the requirements when we select the paper and criteria when we put down the information, to develop the data.

 5. I suggest an English language edit for the revision of the manuscript.

**Answer:** Authors would like say thank to second reviewer. The manuscript has been edited for English language usage by a native British proof-reader of Britannia Proofreading Service.
